# Net power output and thermal efficiency data for single and double flash cycles of Cerro Prieto geothermal power plants

**DOI:** 10.1016/j.dib.2019.104698

**Published:** 2019-10-22

**Authors:** Emilio Hernández Martínez, Patricia Avitia Carlos, José Isaac Cisneros Solís, María del Carmen Prieto Avalos

**Affiliations:** a Escuela de Ciencias de la Ingeniería y Tecnología, Universidad Autónoma de Baja California, Boulevard Universitario #1000, Unidad Valle de las Palmas, Tijuana, B.C., C.P. 21500, Mexico; b Facultad de Ingeniería Mexicali, Universidad Autónoma de Baja California, Boulevard Benito Juárez S/N, Parcela 44, Mexicali, B.C., C.P. 21280, Mexico

**Keywords:** Geothermal power plants, Cerro prieto, Net power output, Thermal efficiency, Well temperature, Separator pressure, Condenser pressure

## Abstract

The data presented below is the thermodynamic simulation and mathematical model development for the single and double flash cycles of Cerro Prieto geothermal power plants. For more insight into analysis thermodynamic, please see “thermodynamic simulation and mathematical model for single and double flash cycles of Cerro Prieto geothermal power plants” [1]. The datasets contained in this paper are thermodynamic simulations obtained in Aspen Hysys software, the data described represents the net power output and thermal efficiency for Cerro Prieto geothermal power plants, located in Mexicali, México. A single flash and double flash cycle have been selected for power generation at this facility. The single flash net power output and the thermal efficiency data includes eight main parameters: Well temperature (°C), Separator pressure (kPa), Condenser pressure (kPa), Turbine's power (kW), Phase Fraction, Mass Flow (kg/h), Energy input (kW) and Energy output (kW). Whereas the double flash net power output and the thermal efficiency data includes eight main parameters: Well temperature (°C), High-pressure separator (kPa), Low-pressure separator (kPa), Condenser pressure (kPa), Turbine's power (kW), Phase Fraction, Mass Flow (kg/h), Energy Input (kW) and Energy output (kW).

**Table:** Specifications table

Subject	Energy
Specific subject area	Energy Engineering and Power Technology
Type of data	Table Figure
How data were acquired	The numerous hydrogeological-geochemical studies made at the Cerro Prieto area have found geothermal fluid temperatures ranging between the ranges of 150 °C–350 °C [[2], [3], [4], [5], [6], [7], [8], [9]]. Thermodynamic Simulation for energy flows were developed and implemented in Aspen Hysys software.
Data format	Raw Filtered
Parameters for data collection	Data was obtained in computer simulations applying the energy and exergy analysis using the laws of thermodynamics.
Description of data collection	An Excel sheet was generated for the compilation of the parameters (State, Temperature, Pressure, Phase Fraction, Mass Flow, Turbine's power, Pump's power, Energy input, Energy output, Net power and Thermal efficiency) generated in the computer simulation
Data source location	Institution: Facultad de Ciencias de la Ingeniería y Tecnología (FCITEC) de la Universidad Autónoma de Baja California (UABC) City/Town/Region: Tijuana/Baja California Country: México Latitude and longitude (and GPS coordinates) for collected samples/data: 32.4356, −116.6751
Data accessibility	With the article Repository name: Thermodynamic simulation and mathematical model for single and double flash cycles of Cerro Prieto geothermal power plants Data identification number: ---- Direct URL to data: https://doi.org/10.17632/9384yj4xg3.5
Related research article	Author's name Emilio Hernández Martínez Title Thermodynamic simulation and mathematical model for single and double flash cycles of Cerro Prieto geothermal power plants Journal Geothermics DOI https://doi.org/10.1016/j.geothermics.2019.101713

**Table:** 

**Value of the Data** • The detailed breakdown of a thermodynamic simulation for a single and double flash cycles are presented, which would enable the analysis and mathematical modelling for similar geothermal power plants at other locations. • This data provide certainty behind the modelling assumptions and the methodology used for a multiple linear regression, the mathematical modelling approach used to predict the net power and the thermal efficiency for single and double flash cycles. • This data provides assistance for the enforcement of an exergoeconomic analysis of single and double flash cycles for Cerro Prieto geothermal power plants. • Data obtained by of a thermodynamic simulation of a single and double flash cycles is presented, which would enable an exergy analysis aimed to propose a modification to the single and double flash-organic Rankine cycles.

## 1 Data

Data reported here concern the net power and the thermal efficiency of Cerro Prieto geothermal power plan, the data has been obtained in Aspen Hysys software, using a compilation of numerous hydrogeological and geochemical studies made to Cerro Prieto power plant. The dataset contains raw and filtered sequencing data obtained through the thermodynamic simulation for the single and double flash cycles of Cerro Prieto geothermal power plants. The data files [reads in Microsoft Excel Worksheet (.xlsx)] were deposited at Mendeley Data under dataset accession https://doi.org/10.17632/9384yj4xg3.5. Information about simulation parameters in Aspen Hysys, parameters for major stages of a single flash power plant operating at optimal pressure and parameters for major stages of a double flash power plant operating at optimal pressure is presented in Table 1, Table 2, Table 3, respectively.

**Table 2:** Important parameters for major stages of a single flash power plant operating at optimal pressure.

State	Pressure (kPa)	Temperature (°C)	Mass flow (kg/s)	Enthalpy (kJ/kg)	Exergy (kW)
1	3972.76	250	420	−14880.12	109998.78
2	650	162.2	420	−14880.12	97709.32
3	650	162.2	337.98	−15291.75	34790.92
4	650	162.2	82.02	−13183.93	62918.40
5	11.50	48.51	82.02	−13710.62	12652.06
6	11.50	48.51	82.02	−15769.79	306.21
7	650	48.54	82.02	−15769.03	376.37
8	650	140.2	420	−15384.96	31598.04

**Table 1:** Simulation parameters in Aspen Hysys.

Parameters	Start	End	Step Size	# Steps
Single Flash Cycles				
Well temperature (°C)	245	255	0.25	41
Separator pressure (kPa)	650	750	2.5	41
Condenser pressure (kPa)	11.5	12.5	0.025	41
Double Flash Cycles				
Well temperature (°C)	315	325	0.5	21
High-pressure separator (kPa)	1050	1200	7.5	21
Low-pressure separator (kPa)	385	395	0.5	21
Condenser pressure (kPa)	11.5	12.5	0.05	21

**Table 3:** Relevant parameters for major stages of a double flash power plant operating at optimal pressure.

State	Pressure (kPa)	Temperature (°C)	Mass flow (kg/s)	Enthalpy (kJ/kg)	Exergy (kW)
1	11356.63	320	480.00	−14439.84	228173.46
2	1200	188.2	480.00	−14439.84	193288.42
3	1200	188.2	305.40	−15178.00	43243.17
4	1200	188.2	174.60	−13148.72	150045.25
5	385	142.4	174.60	−13329.65	114453.79
6	385	142.4	305.40	−15178.00	40738.96
7	385	142.4	277.45	−15375.84	21505.02
8	385	142.4	27.95	−13214.24	19233.93
9	385	142.4	202.55	−13313.73	133687.73
10	11.5	48.51	202.55	−13750.92	30648.04
11	11.5	48.51	202.55	−15769.79	756.20
12	385	48.53	202.55	−15769.35	857.55
13	385	102.5	480.00	−15541.89	17615.86

For this single flash cycle research, a well temperature range of 245 °C–255 °C is established [2]. The optimal separation pressure reported in the bibliography is 100 kPa–1000 kPa [[10], [11], [12], [13]], likewise a pressure range in the separator from 650 kPa to 750 kPa is established for this research. While the condenser pressure is assumed to be in a range from 11.5 kPa to 12.5 kPa, consistent with specialized literature [2,14]. Meanwhile the modelling process of double flash cycle the temperature of the geothermal fluid of Cerro Prieto is considered in between 315 °C and 325 °C; the pressure in the high-pressure separator is established in the ranges from 1050 kPa to 1200 kPa; the pressure in the low-pressure separator is established in the ranges from 385 kPa to 395 kPa; and the pressure in the condenser is established in the ranges from 11.5 kPa to 12.5 kPa [2,15].

## 2 Experimental design, materials, and methods

Firstly, a large part of the data has been collected from specialized articles on geothermal power plants. Then a detailed thermodynamic simulation has been developed for each of the geothermal power plants (single and double flash cycles) to obtain the data to be used in a multiple linear regression analysis.

**Fig. 1:**
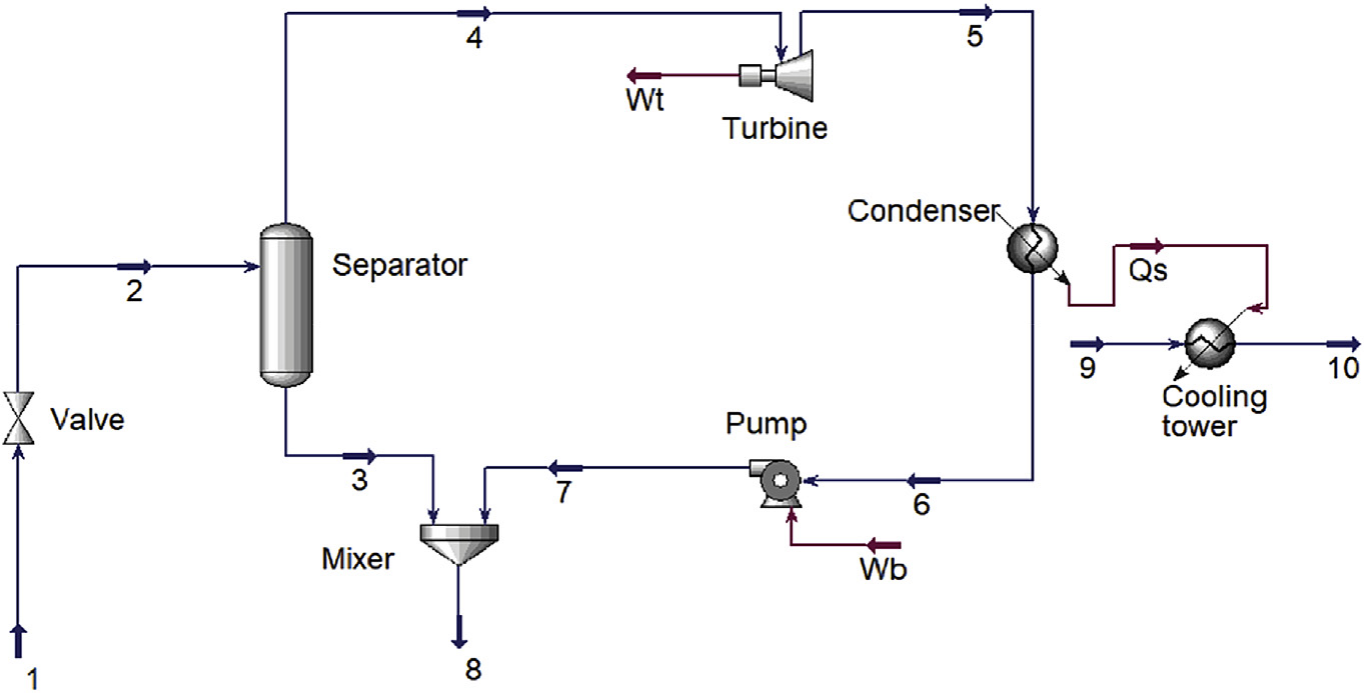
Aspen Hysys diagram of a single flash cycle.

**Fig. 2:**
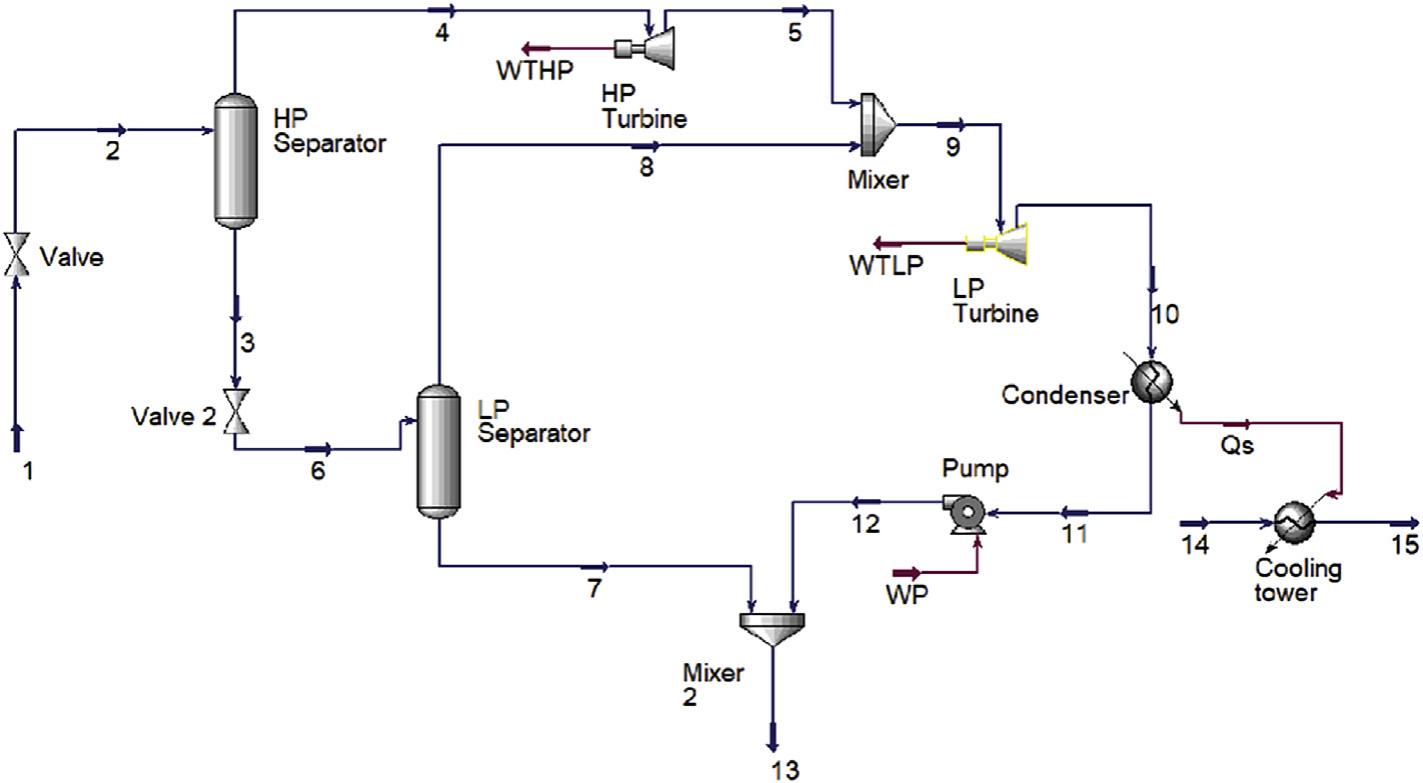
Aspen Hysys diagram of a double flash cycle.

The process diagram for the proposed single flash power cycle was made in Hysys and is shown in Fig. 1. The mass flow rate from the production wells, wellhead temperature, pressure in the separator and pressure in the condenser were used to calculate the remaining parameters of the fluid such as enthalpy and entropy [1]. With reference to Fig. 2, the double flash processes, state 1-state 2 and state 3-state 6, are analysed in the same way as the single flash process. The single flash plant was analysed as described previously in this paragraph. Each process generates a fractional amount of steam which is given by the quality of the two-phase mixture [1].

**Fig. 3:**
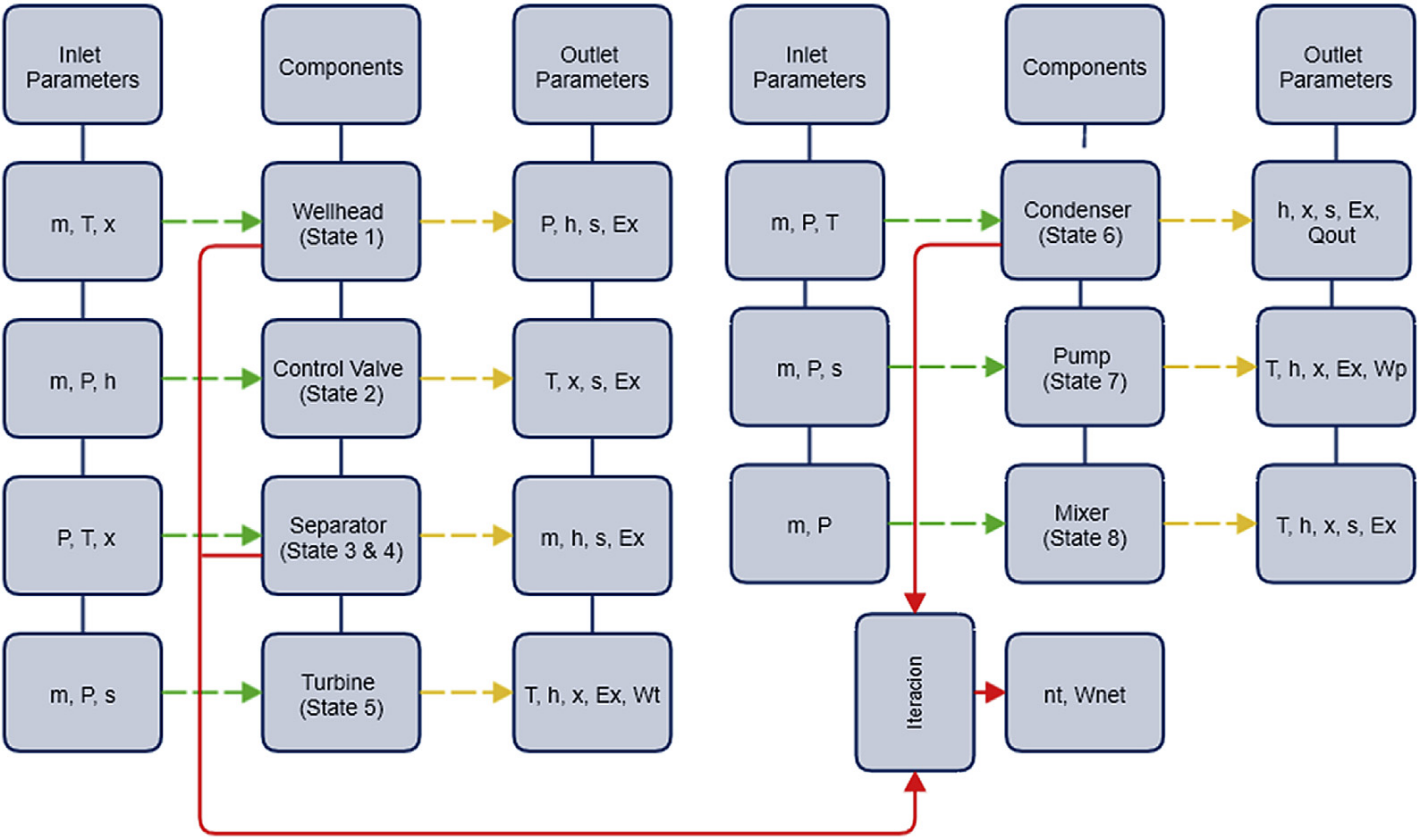
Optimization process flow chart of a single flash cycle.

**Fig. 4:**
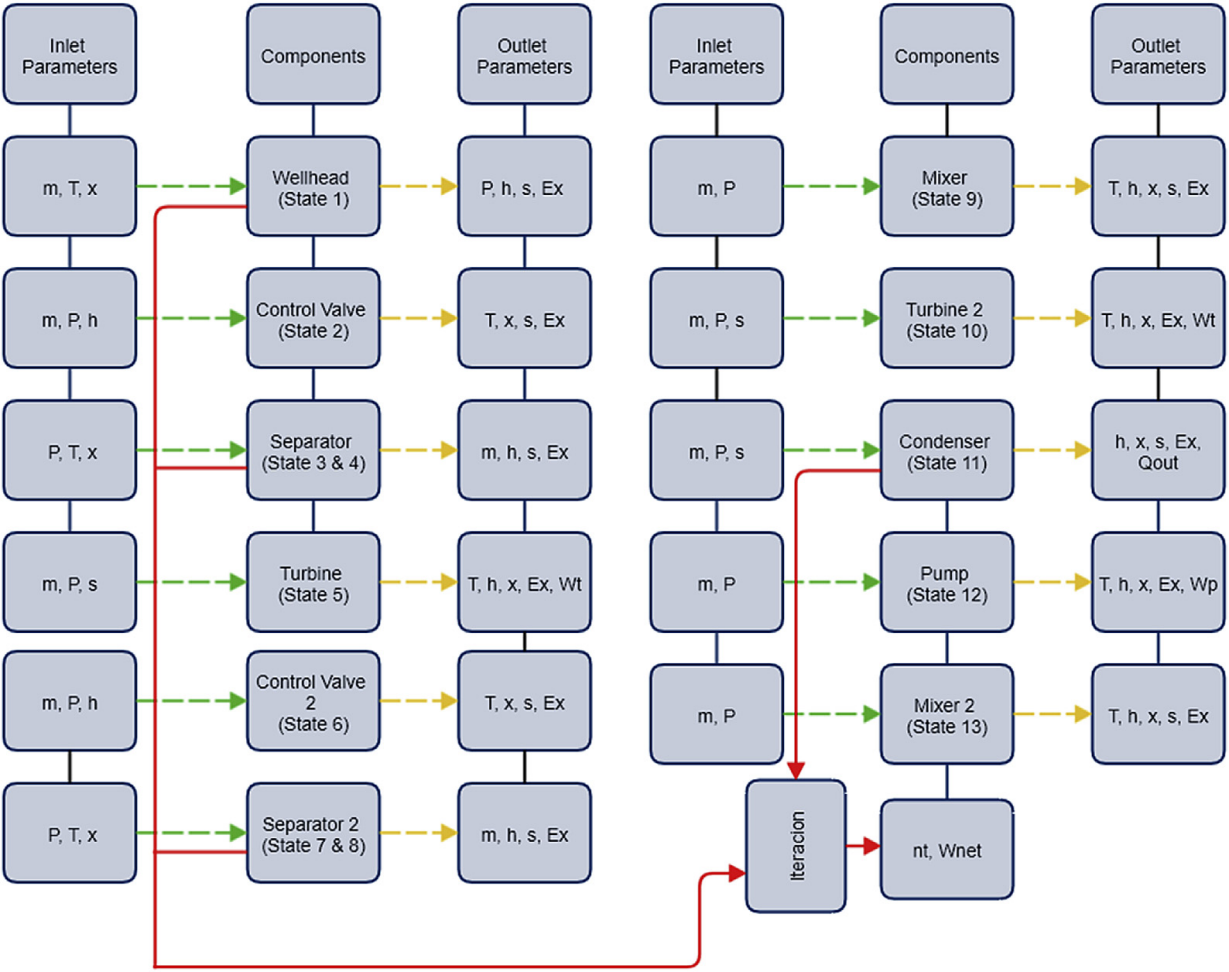
Optimization process flow chart of a double flash cycle.

Fig. 3 illustrates the modelling process of single flash cycle developed in Aspen Hysys. The model follows the flow pattern of a geothermal brine. Fluid enters in each component of the plant and its behaviour is analysed with the related balance equation and specifications from the methodology for energy and exergy analysis. Each component has known and unknown parameters. Meanwhile Fig. 4 illustrates the modelling process of a double flash cycle also developed in Hysys. The modelling process is analysed in the same way as the flash process for the single flash plant previously described was.

## 3 Data processing

The mathematical model is designed to predict the net power and thermal efficiency for single flash cycles, using the independent variables of well temperature, separator pressure and condenser pressure to obtain a mathematical expression that predicts the behaviour of this cycle, which is widely used in the geothermal power plant of Cerro Prieto. As for the double flash cycles, the well temperature, the pressures in the separators and the condenser pressure are used. Based on the aforementioned, an operation interval is established for both cycles as shown below (Table 1):

263,402 simulations were generated, of which 68,921 correspond to the single flash cycle and 194,481 to the double flash cycle. Table 2, Table 3 presents the relevant parameters (pressure, temperature, mass flow rate, enthalpy and exergy) at major stages of the single flash and double flash power plants operating at optimal pressure.

All the collected data was statistically analysed to characterize the mathematical model to predict the net power and thermal efficiency for single flash and double flash cycles. Table 4, Table 5 report statistical indicators which describe the 263,402 simulations were generated at the time of the paper preparation.

**Table 4:** Regression model for double flash cycle.

Dependent variable	r	$R^{2}$	N	p−value
Net power	0.999885	0.999769	68,921	0.0001
Thermal efficiency	0.999628	0.999256	68,921	0.0001

**Table 5:** Regression model for double flash cycle.

Dependent variable	r	$R^{2}$	N	p−value
Net power	0.999861	0.999722	194,481	0.0001
Thermal efficiency	0.999806	0.999612	194,481	0.0001
